# The roles of basophils, TSLP and IL-33 in food allergy following epicutaneous sensitisation

**DOI:** 10.1186/2045-7022-5-S3-O17

**Published:** 2015-03-30

**Authors:** Tomohiro Yoshimoto, Taichiro Muto, Ayumi Fukuoka, Kazufumi Matsushita

**Affiliations:** 1 Hyogo College of Medicine, Nishinomiya, Japan

## 

Cutaneous sensitization with a food antigen before its consumption elicits the development of food allergy. Here we report the site and stage dependent roles of basophils and pro-allergic cytokines, thymic stromal lymphopoietin (TSLP) and IL-33, in a mouse model of food allergy initially sensitized cutaneously with the food antigen. Mice are epicutaneously sensitized with the food antigen ovalbumin (OVA) followed by oral challenge with OVA. Epicutaneously-sensitized mice produce OVA-specific IgE and develop IgE-dependent anaphylaxis after oral challenge. If OVA is given orally before epicutaneous administration, development of food allergy is prevented with *Foxp3* mRNA up-regulation. Thus, our mouse model clearly reflects the “dual-allergen-exposure hypothesis” in which allergic sensitization results from cutaneous food antigen exposure before its consumption. When allergy is induced by epicutaneous sensitization then oral challenge, basophil-depleted or TSLP-receptor-deficient mice do not produce OVA-specific IgE and are protected from oral challenge-induced anaphylaxis. IL-33-deficient mice produce normal levels of OVA-specific IgE, however, IL-33-deficient mice and mice treated with recombinant soluble IL-33 receptor are protected from anaphylaxis. Thus, basophils and TSLP have pivotal roles in Th2 development in the skin during the sensitization phase of food allergy. In contrast, while IL-33 is dispensable for promoting cutaneous antigen sensitization, the cytokine is essential for inducing IgE-dependent anaphylaxis in the gut.

